# Susceptibility of microbes to far-UVC light (222 nm) on spacecraft and cleanroom surfaces

**DOI:** 10.1128/spectrum.02276-25

**Published:** 2025-10-31

**Authors:** Camryn Petersen, Joshua Urbano, Akemi Hinzer, Igor Shuryak, Eric Wang, Raabia Hashmi, Lisa Guan, Manuela Buonanno, David Welch

**Affiliations:** 1 Center for Radiological Research, Columbia University Irving Medical Center, New York, New York, USA; 2 California State Polytechnic University 6647 , Pomona, California, USA; 3 Biotechnology and Planetary Protection Group, Jet Propulsion Laboratory, California Institute of Technology 6469, Pasadena, California, USA; Connecticut Agricultural Experiment Station, New Haven, Connecticut, USA

**Keywords:** planetary protection, antimicrobial, germicidal UV, ultraviolet radiation, microbial reduction, far-UVC, ultraviolet light

## Abstract

Far-UVC light (222 nm) is an emerging decontamination tool with potential for spacecraft assembly applications due to its strong germicidal efficacy and minimal risk to human health. This study evaluated the susceptibility of three UV-resistant microbial species, *Deinococcus radiodurans* and *Bacillus subtilis* as vegetative cells and *Bacillus pumilus* as spores, across eight spacecraft and cleanroom-relevant materials with varying surface properties, including contact angle, reflectivity, and roughness. While *D. radiodurans* demonstrated the highest overall resistance to far-UVC, material properties significantly influenced its inactivation rate (k_1_ [cm^2^/mJ]), and its susceptibility varied significantly by surface. No significant relationship between material properties influencing k_1_ values was observed for *B. subtilis* and *B. pumilus*. Collectively, these findings highlight far-UVC as a robust and practical bioburden reduction tool capable of inactivating resistant microbes regardless of material surface characteristics.

**IMPORTANCE:**

This study builds upon previous research on the inactivation of hardy bacteria and spores using far-UVC light by evaluating the susceptibility of microbes to far-UVC light across a range of surface materials. By focusing on hardy organisms and diverse substrates, this work provides critical insights into the practical applications of far-UVC technology in complex environments. Microbial disinfection is a key component of planetary protection, ensuring that space exploration activities do not inadvertently transport terrestrial organisms to extraterrestrial environments. These findings demonstrate that far-UVC is effective across materials with varying properties and can serve as an effective alternative for bioburden reduction that can be safely operated in occupied spaces.

## INTRODUCTION

The exploration of the solar system and the search for extraterrestrial life are fundamental objectives of modern space missions. Preserving both scientific integrity and extraterrestrial environments between Earth and other celestial bodies during exploration is a pillar of NASA’s Office of Planetary Protection. The Planetary Protection discipline is focused on preventing biological contamination between Earth and other celestial bodies during space exploration—known as forward contamination—and backward contamination, which involves the introduction of potential extraterrestrial life or bioactive molecules to Earth. Maintaining the terrestrial microbial cleanliness of spacecraft is critical to preserving scientific accuracy and protecting planetary ecosystems. As scientific instruments onboard spacecraft become more advanced and sensitive, so too must cleaning efforts to ensure scientific integrity in measuring pristine interplanetary environments, free of contamination from Earth. In crewed missions, microbial reduction modalities are necessary for uncontrolled microbial reservoirs that may increase risks of opportunistic infections and allergenic responses (1, 2). In closed environments such as the International Space Station, microorganisms can persist on hardware, form biofilms that drive corrosion, and biofouling of aerospace alloys, coatings, and fluidic systems (3). Such processes can degrade surfaces, clog tubing, or interfere with instrument function, ultimately reducing spacecraft reliability. Beyond NASA, planetary protection is a globally recognized priority, guided by international policies established by the Committee on Space Research to uphold ethical and scientific standards in space exploration (4).

Since the Viking lander missions in the 1970s, the Biotechnology and Planetary Protection Group at the Jet Propulsion Laboratory (JPL) has developed and implemented methods to monitor and reduce microbial bioburden during spacecraft assembly and launch operations. Traditional decontamination methods used in spacecraft assembly each have their own limitations. Dry heat microbial reduction (DHMR) achieves significant bioburden reduction but requires high temperatures for extended periods, rendering it incompatible with sensitive avionics, optics, and adhesives (5, 6). Chemical wiping with isopropanol (IPA) is widely practiced but cannot consistently access crevices, complex geometries, or multi-layered surfaces and requires significant hands-on time (7). Vapor phase hydrogen peroxide can damage certain polymers, elastomers, and coatings and requires cycle-specific validation for each hardware configuration (8). These approaches present challenges, including material compatibility, logistical constraints, and the need for large-scale sterilization infrastructure. Despite decontamination strategies, humans are the primary source of contamination in cleanroom environments (9).

One emerging approach to aid in decontamination of cleanroom environments is the use of ultraviolet (UV) light, a well-established technology for the inactivation of microorganisms that dates back more than 100 years (10). Conventional germicidal UV, using sources primarily emitting 254 nm, while being highly effective, is limited in application as a result of health risks due to overexposure to human eyes and skin, such as photokeratitis or erythema (11–13). Far-UVC wavelengths in the range of 200 nm–235 nm have limited penetration depth in biological materials compared to longer-wavelength lights such as 254 nm (14–17) due to the light being absorbed by proteins and biomolecules in the stratum corneum of the skin and the outermost surface of the eye (18–20), while still maintaining high efficacy in microbial reduction (21–27). Far-UVC is still capable of damaging the DNA and RNA of bacteria and viruses, as their size is less than 1 µm in diameter. Far-UVC implementation for airborne pathogen inactivation has been a well-documented application (28–31) with its versatility in microbial reduction during spacecraft assembly becoming an emerging tool. Together with its antimicrobial properties, far-UVC’s shorter wavelengths do not damage skin and eyes; this implies that continuous disinfection with far-UVC lamps is possible even when people or workers are present.

Previous studies with far-UVC for planetary protection applications have focused on 222 nm inactivation of hardy vegetative cells and spores of microbial species recovered from NASA spacecraft assembly facilities (26, 32) on 6061 aluminum exclusively. The efficacy of UV light disinfection relies on adequate line-of-sight from the UV light source to microbes, which may be altered if surface properties prevent even exposure. To assess the feasibility of far-UVC as a microbial reduction tool with utility across diverse surfaces in a cleanroom, we evaluated 222 nm bioburden reduction efficacy on different spacecraft and cleanroom materials.

This study examines the efficacy of far-UVC light on three UV-resistant microbial strains across eight different cleanroom facility materials with varying surface properties. The first, *Deinococcus radiodurans,* is a gram-negative, non-sporulating bacterium that withstands various extreme conditions, including cold, dehydration, vacuum, strong oxidants, and radiation (33, 34). Cells are approximately 2 µm in diameter (35). *D. radiodurans* possesses an efficient DNA damage repair pathway and a high-efficiency antioxidant defense system (35–37) and has been selected for multiple simulated space environment studies due to its extremophile qualities (33, 34). In our previous dose-response study, *D. radiodurans* proved to be the most resistant of the tested species on 6061 aluminum, with a k_1_ value of 0.021 **±** 7.2 × 10^−3^ cm^2^/mJ (32). The second selected vegetative cell species is *Bacillus subtilis,* a gram-positive, aerobic spore former. While this species can sporulate, only vegetative cells were tested in this study. Vegetative *B. subtilis* cells are rod-shaped and measure 2 µm–6 µm long and approximately 1 µm in diameter (38). *B. subtilis* has shown high tolerance to extreme environments, and it is a commonly isolated bacterium in cleanroom environments, making it an organism of interest for planetary protection and space microbiology. This species has shown capabilities of surviving in Mars analog soil, therefore posing a risk for forward contamination (39, 40), and it has been shown to survive vapor hydrogen peroxide exposure (41). *Bacillus pumilus* SAFR-032 spores were tested in this study. *B. pumilus* SAFR-032 is a gram-positive, aerobic, spore-former isolated from the spacecraft assembly facility at JPL (42), capable of withstanding vapor hydrogen peroxide exposure, resistance to desiccation, and UV exposure (39–41). *Bacillus pumilus* spores are approximately 1 µm in length and 0.5 µm in diameter (43). Bacterial endospores create a challenging issue for planetary protection due to their tremendous resilience to environmental stressors compared to vegetative cells (44), which can potentially compromise the scientific integrity of space missions if they contaminate sensitive instruments or samples of interest.

In addition to selecting radioresistant microbial strains, this study examines the influence of surface properties—specifically roughness, wettability (contact angle [CA]), and reflectivity—on the susceptibility of microbes to far-UVC irradiation. Surface roughness has been shown to influence far-UVC inactivation of microbes, as rougher surfaces may create shading that results in a loss of inactivation efficiency (45). Wettability, indicated by contact angle measurements, also plays a critical role. Hydrophobic surfaces tend to cause liquid droplets to bead up, which could lead to increased cell piling and potential shadowin g effects that diminish 222 nm exposure. Conversely, hydrophilic surfaces cause water droplets to spread into thinner films, reducing piling and minimizing shadowing effects as the droplets dry (46, 47). Reflectivity can influence the efficacy of UV decontamination methods, as surfaces with higher reflectivity may increase the effective dose received by microorganisms (48), impacting survival and thus inactivation rates. By evaluating these factors across various spacecraft- and cleanroom-relevant materials, this study aims to elucidate how surface characteristics may affect microbial susceptibility to far-UVC exposure, thereby informing the development of more effective planetary protection protocols for a variety of disinfection scenarios.

## MATERIALS AND METHODS

### Preparation of vegetative bacteria cells

*B. subtilis* (DSM type strain 10, DSMZ-German Collection of Microorganisms and Cell Cultures GmbH, Germany) vegetative cells were grown on tryptic soy agar (R455004, REMEL Inc., San Diego, CA) at 32°C for 48 h. A bacterial solution was prepared by inoculating a colony in tryptic soy broth (TSB, t8907, Sigma Aldrich, St. Louis, MO) while shaking at 37°C overnight. One milliliter of the bacterial solution was then added to 20 mL of fresh TSB and allowed to grow until reaching an exponential phase, identified with an optical density measurement of 0.600–0.800 for OD_600_ using an Eppendorf Biophotometer D30 (Eppendorf, Enfield, CT). *D. radiodurans* (DSM 20539) cells were grown on tryptone glucose yeast extract (TGY), consisting of 5% tryptone (211705, Thermo Fisher, Fair Lane, NJ), 5% yeast extract (bp1422-500, Thermo Fisher), 1% glucose (A16828.36, Thermo Fisher), 1% dipotassium phosphate (p288-500, Thermo Fisher), and 20% agar (05040-1KG, MilliporeSigma, Burlington, MA), at 32*°*C for up to 72 h. A *D. radiodurans* solution was prepared by inoculating a *D. radiodurans* colony from the TGY plate into 40 mL TGY broth and shaking at 32*°*C for 24 h.

### Preparation of *Bacillus* spores

*B. pumilus* spore cultures were prepared with Difco sporulation medium (44) SAFR-032 in sterile 100 mm Petri dishes (Corning, USA). Contrast phase microscopy was utilized to confirm spore yield. Spores were then harvested into sterile, deionized water, washed with 1 M KCl/0.5 M NaCl, followed by 50 mM Tris-HCl, pH 7.5, with lysozyme (50 µg/mL), and finally 1M NaCl solutions. These steps were followed by four washes in cold, sterile deionized water, a heat shock at 80°C for 15 minutes, and two more water washes.

### UV lamps

Krypton-chlorine (KrCl) excimer lamps emitting principally at 222 nm were used for exposures. The lamps (High Current Electronics Institute, Tomsk, Russia) emit through a 6,000 mm^2^ exit window (49). Consistent with our previous study on far-UVC germicidal efficacy for planetary protection applications (26, 32), a custom bandpass filter was used with the KrCl lamp to reduce emissions other than the 222 nm peak. The spectral irradiance of the lamp with the optical filter is included in the Supplemental Materials. Optical power measurements at JPL were performed using an 818-UV/DB low-power UV-enhanced silicon photodetector with an 843-R optical power meter (Newport, Irvine, CA). Optical power measurements at Columbia University used a Hamamatsu C9536 power meter with an H9535-222 sensor head (Hamamatsu Corporation, Bridgewater, NJ). All exposures were performed with an irradiance of 0.5 mW/cm^2^. To deliver different radiant exposures (doses), the exposure time was changed.

### Bacterial exposures on selected surfaces

Exposures of bacteria were conducted on coupons made of eight proxy spacecraft assembly facility surfaces. Coupons were cut 1” by 1” and sterilized by submersion in 70% ethanol for 30 minutes, followed by exposure to 254 nm emitted in a laminar flow hood for 1 h on each side. Sterility was checked by incubating a coupon in TSB for up to 72 h at 32*°*C and assessing for turbidity. A minimum of three biological replicates was done per species.

Aluminum 6061 (6061-T6, onlinemetals.com, Seattle, WA) was utilized as a proxy spacecraft surface, as it is commonly used in spaceflight applications. We have used this material in previous studies (26, 32) to determine the initial susceptibility of relevant microbes to 222 nm light. For the remainder of this paper, we refer to this material simply as “aluminum.”

Stainless steel 304 2B (6824, onlinemetals.com, Seattle, WA) is a widespread material used in space applications due to its rust resistance. Stainless steel comes in various alloys and surface finishes; however, we have focused on 304 2B, as it is the most used for spaceflight applications. This finish grade is unpolished with only partially sealed grain boundaries, which have been shown to support the growth of microorganisms (50). For the remainder of this paper, we refer to this material simply as “stainless steel.”

Multi-layer insulation (JPL, Pasadena, CA) is frequently used on spacecraft for thermal insulation and is comprised of several layers of aluminized Mylar. For the remainder of this paper, we refer to this material simply as “MLI.”

Aeroglaze Z306 (Z/30011434, Socomore, Rhome, TX) paint was applied to aluminum coupons. This polyurethane coating is primarily used for space operations where coatings must have low outgassing and high thermal absorptivity characteristics (51). For the remainder of this paper, we refer to this material simply as “black paint.”

Chemfilm on aluminum (Precision Anodizing & Plating, Anaheim, CA) is used to prevent corrosion and can also be used as a paint primer. For the remainder of this paper, we refer to material simply as “chemfilm.”

Clear polyvinyl chloride (87545K121, mccaster.com, Robbinsville, NJ) is used to form soft walls for cleanroom tents, which are used during launch operations. For the remainder of this paper, we refer to this material simply as “PVC.”

Kapton tape (KPT-1, kaptontape.com, Torrance, CA) is the most common variation of polyimide film in space instruments and electronics due to its ability to remain stable across an extreme range of temperatures. Kapton tape was fixed to an aluminum coupon during testing for ease of handling. For the remainder of this paper, we refer to this material simply as “polyimide.”

Formica laminate (949, formica.com, Cincinnati, OH) is a cleanroom flooring material and was included in this study to help understand the efficacy of applying far-UVC for microbial reduction of spacecraft assembly facilities. For the remainder of this paper, we refer to the material simply as “laminate.”

**Fig 1:**
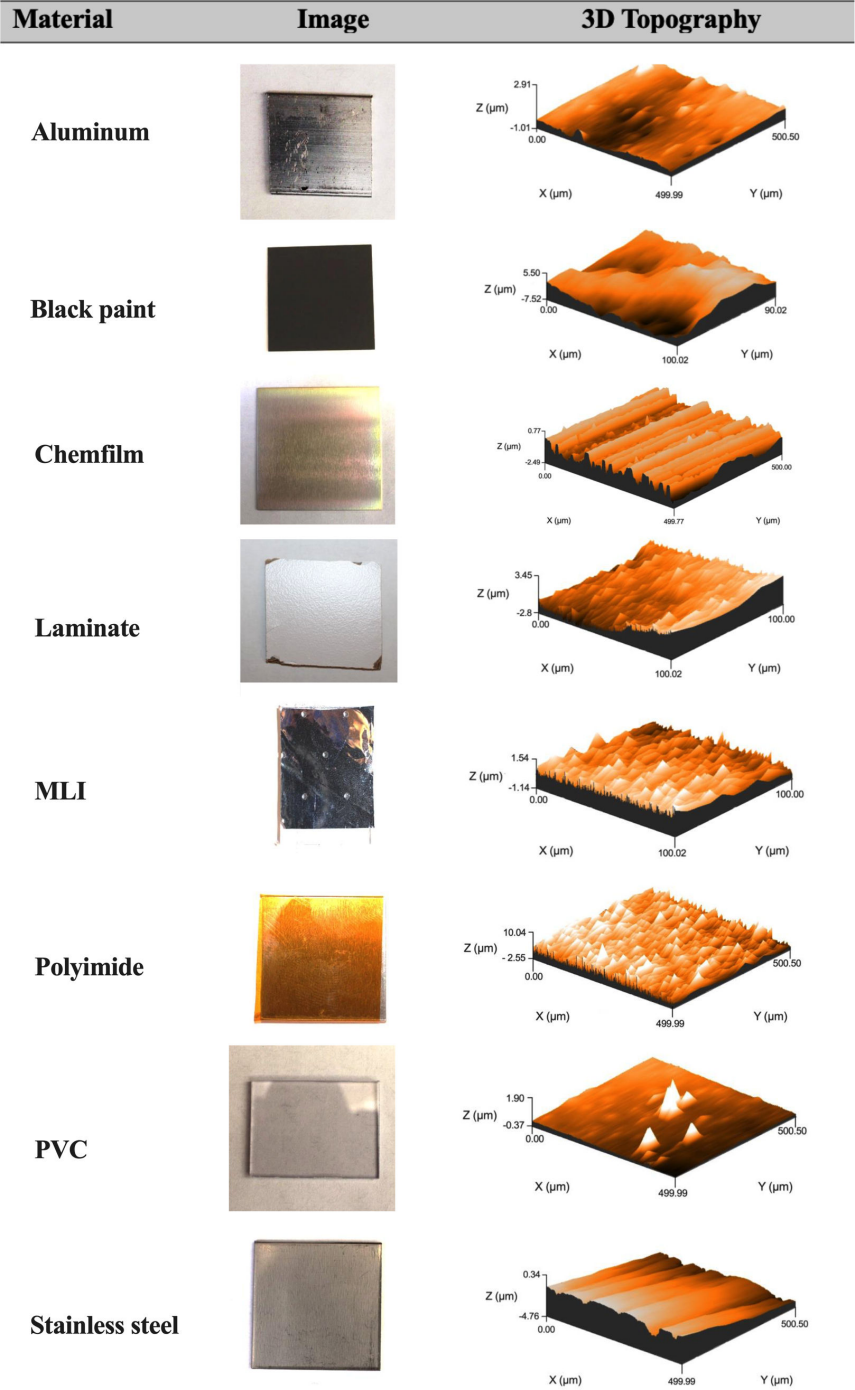
Photos of tested material surfaces and their corresponding three-dimensional (3D) topography. *Z*-axis labels represent the surface height range. Some 3D scan lengths were limited to 100 µm due to data point constraints.

Photographs of each of the test materials are shown in Fig. 1.

For testing, coupons were seeded with 10 separate 10 µL aliquots of bacterial solution containing a concentration of 10^6^–10^7^ colony forming units (CFU) mL^−1^ uniformly across the coupon, totaling 100 µL per coupon. The solution on the coupons was then allowed to dry in sterile Petri dishes inside a biosafety cabinet. Once dry, the coupons were then directly exposed to 222 nm filtered KrCl lamp output over a range of radiant exposures.

Vegetative cells were recovered immediately following exposure by submerging the coupon in 10 mL of 1× phosphate-buffered saline and vortexing for 30 s to remove the bacteria from the surface. Spores were recovered from coupon surfaces by pipetting 200 µL of sterile 10% polyvinyl alcohol (PVA) solution on the coupon and allowing it to dry in a laminar flow hood at room temperature for 1.5–3 h. The PVA was then peeled off using sterile forceps (52, 53) and dissolved in sterile 50 mL conical tubes containing 15 mL of water.

Survival of microbes was determined by direct plating post-exposure using the pour plate method. Bacterial solutions of 1 mL were serially diluted and plated on their respective media. Dissolved spore PVA solution was serially diluted and subsequently plated on its respective media. Plates were incubated at 32°C and colonies were counted at 72 h.

### Material properties

#### Material roughness

The surface roughness of the samples was characterized using the KLA Tencor P17 stylus profilometer equipped with a 2 µm tip radius and 60° cone angle. Measurements were performed in triplicate with a 20 µm/s scan rate and a 500 µm scan line with a force of 2 mg.

#### Contact angle

The contact angle of various materials was measured by aliquoting 10 5 µL drops of deionized water on the material surface (54). The side profile of the drops on the materials was captured using a Canon EOS Rebel T7 (Canon USA) for processing. No image treatment was done to further process images before determining contact angle. The angle was determined using open-source software ImageJ and a contact angle plugin (55). A minimum of 10 replicate measurements were collected.

#### UV reflectivity

The UV reflectivity of the tested materials was evaluated using an experimental setup and protocol established by Ma et al. (24). A filtered 12 W, 222 nm KrCl excimer lamp module (USHIO America, Item #9101711, Cypress, CA) was employed as the UV source. The reflected irradiance from the material surfaces was measured using a calibrated radiometer (UIT2400 Handheld Light Meter for 222 nm, Ushio America, Cypress, CA). The radiometer was positioned at a fixed angle relative to the incident beam to standardize measurements across all samples. The complete experimental configuration, including source positioning, detection angles, and material placement, is illustrated in Fig. 1 of the study by Ma et al. (24). A minimum of 10 replicate measurements were collected.

### Data analysis

Bacterial survival fraction was expressed as the proportional reduction in colony-forming units per milliliter, relative to the unexposed controls for a given experiment. The dose-response relationship was modeled using a two-phase bi-exponential function, a standard approach in disinfection and pathogen inactivation analyses (56). This model accounts for heterogeneous microbial populations by incorporating two distinct exponential populations: the susceptible fraction and the resistant subpopulation:


(1) $$S=(1-f)e^{{-k}_{1}D}+fe^{{-k}_{2}D}$$

Here, *S* represents the surviving fraction of the microorganism, while *D* denotes the radiant exposure dose in millijoules per square centimeter. The term (1 – *f*) corresponds to the proportion of the susceptible population, whereas *f* represents the fraction of the more resistant subpopulation. The inactivation kinetics of both populations follow exponential dose-response curves, characterized by the parameters k_1_ and k_2_ (square centimeters per millijoule), which define the rate of inactivation for the susceptible and resistant fractions, respectively. Matlab (MathWorks, Natick, MA) was used to fit the data to the model and determine estimates for each variable. When estimates for parameters failed to converge, notably due to poor fitting in the tail region of the curve, the value for k_2_ was set to 0 to allow for the convergence of other parameters.

A multiple linear regression model was used to evaluate the relationship between material roughness (Ra), contact angle (θ), and reflectivity (R) as predictor variables, and the k_1_ value as the dependent variable in one model, and the *f* value as the dependent variable in another. The regression model was formulated as follows:


(2) $$k_{1}=\beta_{0}+\beta_{1}Ra+\beta_{2}\theta +\beta_{3}R+\epsilon$$

where β_0_ is the intercept, β_1_, β_2_, and β_3_ are the regression coefficients for each predictor variable, and ϵ represents the residual error. The model was fitted using ordinary least squares regression. Variance inflation factor was utilized to detect multicollinearity. Normality and homoscedasticity were checked through residual and Q-Q plots. The goodness of fit was evaluated by the coefficient of determination (*R*^2^), and predictor significance was indicated by *P*-value. Multiple linear regression analysis was conducted using Prism 10.3.1 (GraphPad Software Inc.) at a significance level of α = 0.05.

## RESULTS

### Cell susceptibility across materials

**Fig 2:**
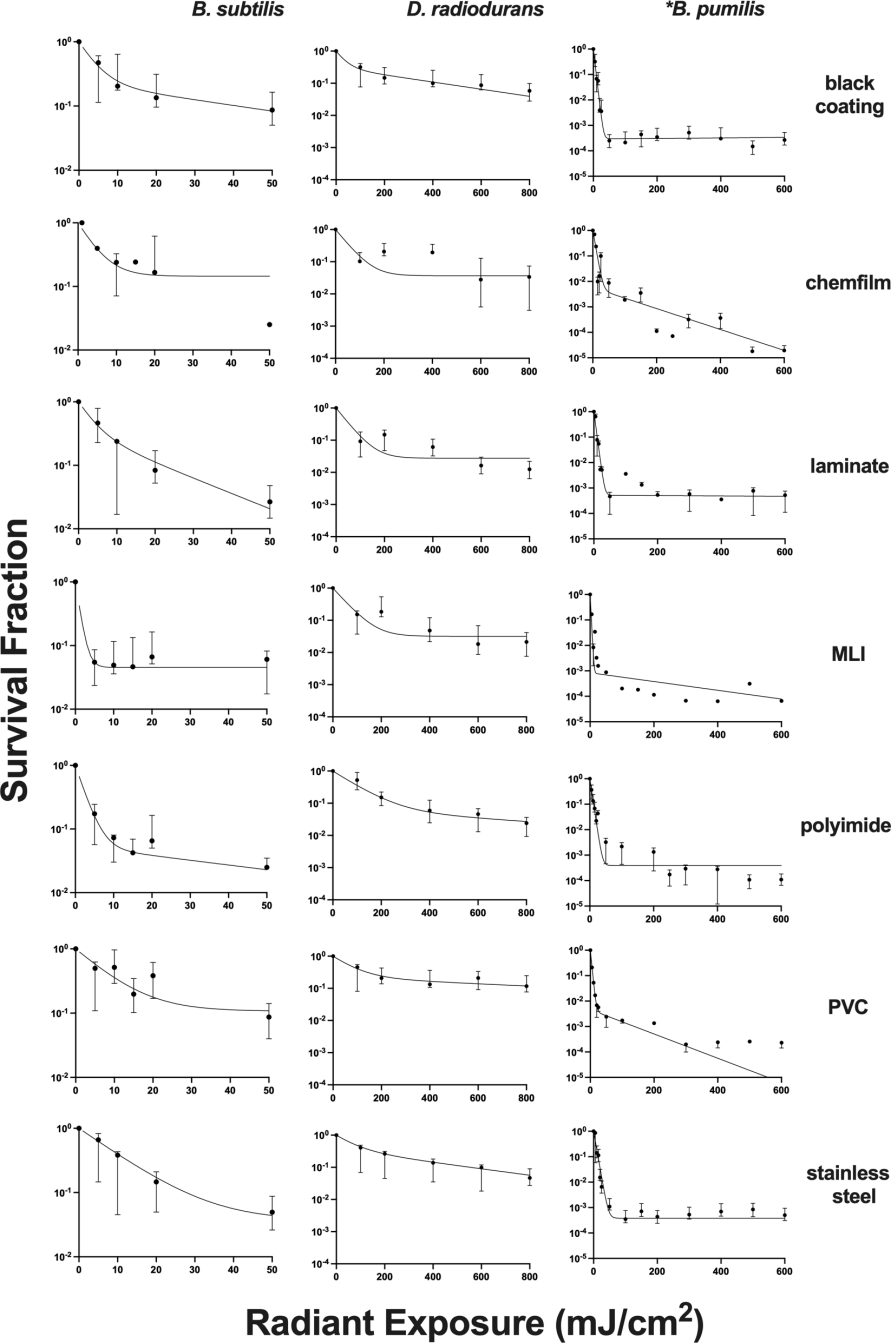
The survival fraction of bacteria exposed to 222 nm light on different materials is plotted for each bacterial species tested (average ± SEM). The best-fit line for the bi-exponential response model in equation 1 is included in each plot. The asterisk indicates the species tested as a spore.

The results of bacterial susceptibility to far-UVC exposure on relevant spacecraft facility materials are presented in Fig. 2. The data used to generate the plots in Fig. 2 can be found in Table S1. Table 1 summarizes the susceptibility estimates and resistant fraction estimates for all microbes and materials based on the fit of the survival data to equation 1. The corresponding best-fit lines from the modeling results are also displayed in Fig. 2.

**TABLE 1:** Characterization of model parameters for bacterial survival fractions for 222 nm light exposure across materials for each tested bacterial species

Species	Material	k_1_ ± SE (cm^2^/mJ)	k_2_ ± SE (cm^2^/mJ)	f ± SE
*B. pumilus* ^*b*^	Aluminum^*a*^	0.34 ± 0.015	3.2 × 10^−3^ ± 6.1x10^−3^	1.0 × 10^−4^ ± 2.0x10^−5^
	Black coating	0.17 ± 9×10^−3^	0 ± 0	3.8 × 10^−4^ ± 9.0x10^−3^
	Chemfilm	0.25 ± 0.01	2.4 × 10^−4^ ± .084	2.9 × 10^−4^ ± 7.0x10^−3^
	Laminate	0.17 ± 0.013	9.4 × 10^−3^ ± .084	5.7 × 10^−3^ ± .031
	MLI	0.22 ± 0.017	1.5 × 10^−4^ ± .097	5.2 × 10^−4^ ± .015
	Polyimide	0.19 ± 5.0×10^−3^	0 ± 0	3.9 × 10^−4^ ± 4.0x10^−3^
	PVC	0.30 ± 0.01	0.011 ± 0.052	4.6 × 10^−3^ ± 9.0x10^−3^
	Stainless steel	0.56 ± 0.14	4.0 × 10^−3^ ± 1.5	8.4 × 10^−4^ ± .033
*B. subtilis*	Aluminum^*a*^	0.57 ± 0.23	0.045 ± 0.14	0.058 ± 0.10
	Black coating	0.98 ± 0.013	0 ± 0	0.037 ± 0.048
	Chemfilm	0.26 ± 0.14	0.020 ± 0.032	0.23 ± 0.21
	Laminate	0.25 ± 0.14	0 ± 0	0.15 ± 0.070
	MLI	0.27 ± 0.21	0.056 ± 0.065	0.34 ± 0.46
	Polyimide	0.43 ± 0.068	0.017 ± 0.032	0.054 ± 0.040
	PVC	0.13 ± 0.04	0 ± 0	0.11 ± 0.089
	Stainless steel	0.93 ± 0.82	0 ± 0	0.045 ± 0.022
*D. radiodurans*	Aluminum^*a*^	0.021 ± 7.2×10^−3^	1.8 × 10^−3^ ± 1.0x10^−3^	0.18 ± 0.097
	Black coating	0.14 ± 5.0×10^−3^	0 ± 0	0.36 ± 0.15
	Chemfilm	0.029 ± 0.026	0 ± 0	0.30 ± 0.16
	Laminate	0.021 ± 3.0×10^−3^	0 ± 0	0.037 ± 0.023
	MLI	0.022 ± 2.0×10^−3^	0 ± 0	0.027 ± 0.012
	Polyimide	0.11 ± 4.0×10^−3^	4.0 × 10^−3^ ± 1.2x10^−3^	0.069 ± 0.19
	PVC	0.013 ± 7.0×10^−3^	7.9 × 10^−4^ ± 2.0x10^−3^	0.22 ± 0.19
	Stainless steel	0.019 ± 3.0×10^−3^	0 ± 0	0.032 ± 0.015

^*a*^ Data for exposures on aluminum are from previously published results (32).

^*b*^ *B. pumilus* was tested as spores.

Of the three cells tested, *D. radiodurans* exhibited the highest resistance to UVC light with lower k_1_ across materials. A multiple linear regression analysis assessed the influence of contact angle, reflectivity, and roughness on *D. radiodurans* k_1_ values across different materials. The independent variables of the model (contact angle, reflectivity, roughness) for *D. radiodurans* were statistically significant (*F*(3,4) = 10.25, *P* = 0.0239), suggesting that surface properties influenced its resistance to 222 nm light. Among the predictors, contact angle (β = 2.73 × 10^−4^, *P* = 0.0396), reflectivity (β = 1.72 × 10^-4^, *P* = 0.0243), and roughness (β = 0.0120, *P* = 0.044) were all significant contributors to k_1_ variability. Notably, *D. radiodurans* was most sensitive on black coating and polyimide (k_1_ = 0.14 **±** 5.0 × 10^−3^ and 0.11 **±** 4.0×10^-3^ cm^2^/mJ, respectively), both of which had low reflectivity values (3.7 ± 0.75 and 4.2 ± 0.53%, respectively); in addition, polyimide had the lowest contact angle and surface roughness (64 ± 7.4 and 0.14 ± 0.034 µm, respectively). *D. radiodurans* proved to be most susceptible to 222 nm light on PVC, with a k_1_ of 0.013 **±** 7.0 × 10^−3^ cm^2^/mJ.

For *B. subtilis* and *B. pumilus*, none of the variables tested in the multiple linear regression model reached statistical significance. With regard to *B. subtilis,* surface contact angle, reflectivity, and roughness did not significantly affect k_1_ values (*F*(3,4) = 0.148, *P* = 0.926), explaining 10.0% of the variance (*R*^2^ = 0.100). Although *B. subtilis* was the most susceptible to 222 nm across materials, k_1_ values on PVC (*P* = 0.170) did not differ significantly from other surfaces, suggesting relatively uniform susceptibility. This species was most susceptible on black coating, with a k_1_ of 0.98 **±** 0.013 cm^2^/mJ. Conversely, it was least susceptible on PVC, with a k_1_ value of 0.13 **±** 0.04 cm^2^/mJ.

Similarly, for *B. pumilus,* the regression model was not statistically significant (*F*(3,4) =0.158, *P* = 0.919), explaining 10.6% of the variance (*R*^2^ = 0.106). None of the surface properties showed a significant effect on k_1_ values (*P* > 0.54). *B. pumilus* spores were most resistant to far-UVC on aluminum, with a k_1_ of 0.021 ± 7.2 × 10^-3^ cm^2^/mJ, and least resistant on stainless steel (k_1_ = 4.0 × 10^-3^ ± 1.5).

### Material properties

Material properties are displayed in Table 2. Most materials exhibit moderate hydrophobic behavior, with contact angles between 70° and 90°. Chemfilm was the only material with a measured contact angle (CA, θ) > 90°, indicating it is the most hydrophobic of the materials tested (CA = 100 ± 9.1°). Aluminum had a CA of 88 ± 9.3° but was still moderately hydrophobic. Additionally, aluminum had the second-highest reflectivity value of 32 ± 0.50%. The most hydrophilic material tested was polyimide, where θ < 70° (CA = 64 ± 7.4°). Laminate had the highest roughness (Ra = 0.34 ± 0.034 µm), whereas MLI was the smoothest (0.05 ± 0.040 µm) and most reflective (66 ± 0.76%). Black coating and PVC were the least reflective materials (3.7 ± 0.75% and 3.9 ± 0.35%, respectively).

**TABLE 2:** Surface properties of spacecraft-relevant materials^*a*^

Material	Contact angle (°)	Reflectivity (%)	Roughness (µm)
Aluminum	88 ± 9.3	32 ± 0.50	0.34 ± 0.034
Black coating	79 ± 7.6	3.7 ± 0.75	0.38 ± 0.037
Chemfilm	100 ± 9.1	26 ± 1.2	0.38 ± 0.035
Laminate	74 ± 3.3	10 ± 3.28	0.85 ± 0.12
MLI	77 ± 4.5	66 ± 0.76	0.05 ± 0.040
Polyimide	64 ± 7.4	4.2 ± 0.53	0.14 ± 0.020
PVC	87 ± 5.6	3.9 ± 0.35	0.14 ± 0.034
Stainless steel	80 ± 4.5	29 ± 1.4	0.31 ± 0.025

^*a*^ Reflectivity refers to the material surface’s reflectance of 222 nm light.

## DISCUSSION

This study examined the susceptibility of UV-resistant microbial species exposed to 222 nm far-UVC irradiation across a range of spacecraft-relevant materials. Our findings highlight both the potential of far-UVC as an effective bioburden reduction strategy and the critical role that material properties play in modulating microbial inactivation.

Among the tested organisms, *D. radiodurans* demonstrated the greatest resistance to far-UVC exposure, consistent with its well-documented extremophile characteristics and robust DNA repair (36) mechanisms. The multiple linear regression analysis revealed that, for *D. radiodurans,* material properties such as roughness, contact angle, and reflectivity were statistically significant predictors of k_1_ values (*F*(3,4) =10.25, *P* = 0.0239) and explained 88.5% of the variance in k_1_ (*R*^2^ = 0.885). Thus, for these studies on *D. radiodurans*, greater contact angle, higher reflectivity, and increased surface roughness were each associated with higher k_1_ values, suggesting a decrease in susceptibility on more hydrophobic, reflective, and rough surfaces. In contrast, no such relationship was observed for *B. pumilus* or *B. subtilis*, where the regression models were not statistically significant (*P* = 0.919 and *P* = 0.926, respectively). These results from *D. radiodurans* may suggest that variations in surface structure can influence inactivation rates by altering cell distribution or the UV dose experienced by a cell. *D. radiodurans* was more susceptible on black coating and polyimide, both materials with low reflectivity and contact angles, suggesting that more hydrophilic, low-reflective surfaces may enhance far-UVC susceptibility. These reflectivity results are somewhat surprising, since reflective surfaces would be expected to enhance the total UV fluence upon each cell. We point out that the outlier k1 values calculated for *D. radiodurans*, with k_1_ for black paint and polyimide almost an order of magnitude larger than k_1_ values on other materials, could have skewed this analysis. A future study examining each of these material properties independently could help untangle their relative importance. Overall, these results indicate that exposure to 222 nm light on select materials may influence the inactivation of highly resistant microbial species, such as *D. radiodurans*. Although these findings should be interpreted with some caution due to the limited number of microbes and materials tested in this study, they demonstrate that surface characteristics could be an important factor in some decontamination scenarios using far-UVC radiation.

While the material property effects on k_1_ were statistically significant for *D. radiodurans*, the practical relevance for planetary protection is limited, since *D. radiodurans* has not been previously isolated directly from spacecraft assembly facilities (39, 57). Its inclusion in studies serves as a model for extreme microbial resistance scenarios. In these instances, we expect that far-UVC will not be the singular decontamination method. The lack of a significant relationship between surface properties and k_1_ values for *B. pumilus* and *B. subtilis* is more representative of the spacecraft bioburden contaminants, suggesting that in real-world applications, differences in properties among common spacecraft assembly cleanroom materials do not substantially influence far-UVC efficacy against microbes. Additionally, although *B. subtilis* and *B. pumilus* differ considerably in size, their survival across materials was similar, suggesting that cell size does not influence far-UVC susceptibility under the conditions tested. These observations support far-UVC light as a promising engineering control to inactivate microbes within occupied spaces, including NASA spacecraft facilities.

Material independence is a valuable advantage, as traditional decontamination techniques such as DHMR, IPA wiping, and hydrogen peroxide vapor sterilization often require material compatibility assessments or extensive handling protocols. In contrast, far-UVC has demonstrated efficacy of disinfection across diverse spacecraft-relevant materials, which allows for flexible, broad-spectrum application without the need to adjust protocols for specific surface types. Other recent studies have similarly demonstrated that far-UVC effectively inactivates a broad range of clinically relevant and antibiotic-resistant bacteria on various materials, including fabrics and hard surfaces, underscoring its potential for diverse disinfection applications beyond spacecraft contexts (58–60). While there are few reports on possible negative effects on materials from far-UVC exposure, studies on both common bus (61) and aircraft (62) materials mainly noted color changes in some polymeric materials, using exposures of 290 J/cm^2^ or 108 J/cm^2^ to simulate 10 years of exposure for bus and aircraft materials, respectively. Future studies analyzing the effects of repeated far-UVC exposure upon materials could also be helpful to evaluate possible adverse effects to the integrity and function of spacecraft-relevant materials.

Collectively, these findings highlight far-UVC as a robust and practical bioburden reduction tool capable of inactivating resistant microbes regardless of material surface characteristics. Although the influence of material properties on extremophiles, like *D. radiodurans*, warrants further mechanistic studies, the broader trend of material independence supports the versatility and operational value of far-UVC in spacecraft assembly facilities.

## SUPPLEMENTAL MATERIAL

Supplemental Material spectrum.02276-25-s0001.docx Fig. S1 and Table S1.

ASM does not own the copyrights to Supplemental Material that may be linked to, or accessed through, an article. The authors have granted ASM a non-exclusive, world-wide license to publish the Supplemental Material files. Please contact the corresponding author directly for reuse.
