# Supplementary material for: Susceptibility of microbes to far-UVC light (222 nm) on spacecraft and cleanroom surfaces
Source: Microbiol Spectr. 2025 Oct 31;13(12):e02276-25. doi: 10.1128/spectrum.02276-25 (PMC12671110; doi:10.1128/spectrum.02276-25)

**Susceptibility of microbes to far-UVC light (222 nm) on spacecraft and cleanroom surfaces**

Camryn Petersen^1^, Joshua Urbano^3^, Akemi Hinzer^2^, Igor Shuryak^1^, Eric Wang^1^, Raabia Hashmi^1^, Lisa Guan^2^, Manuela Buonanno^1^, and David Welch^1*^

^1^ Center for Radiological Research, Columbia University Irving Medical Center, New York, NY, USA

^2^ Biotechnology and Planetary Protection Group, Jet Propulsion Laboratory, California Institute of Technology, Pasadena, CA, USA

^3^ California State Polytechnic University, Pomona, CA, USA

*Supplemental Materials*


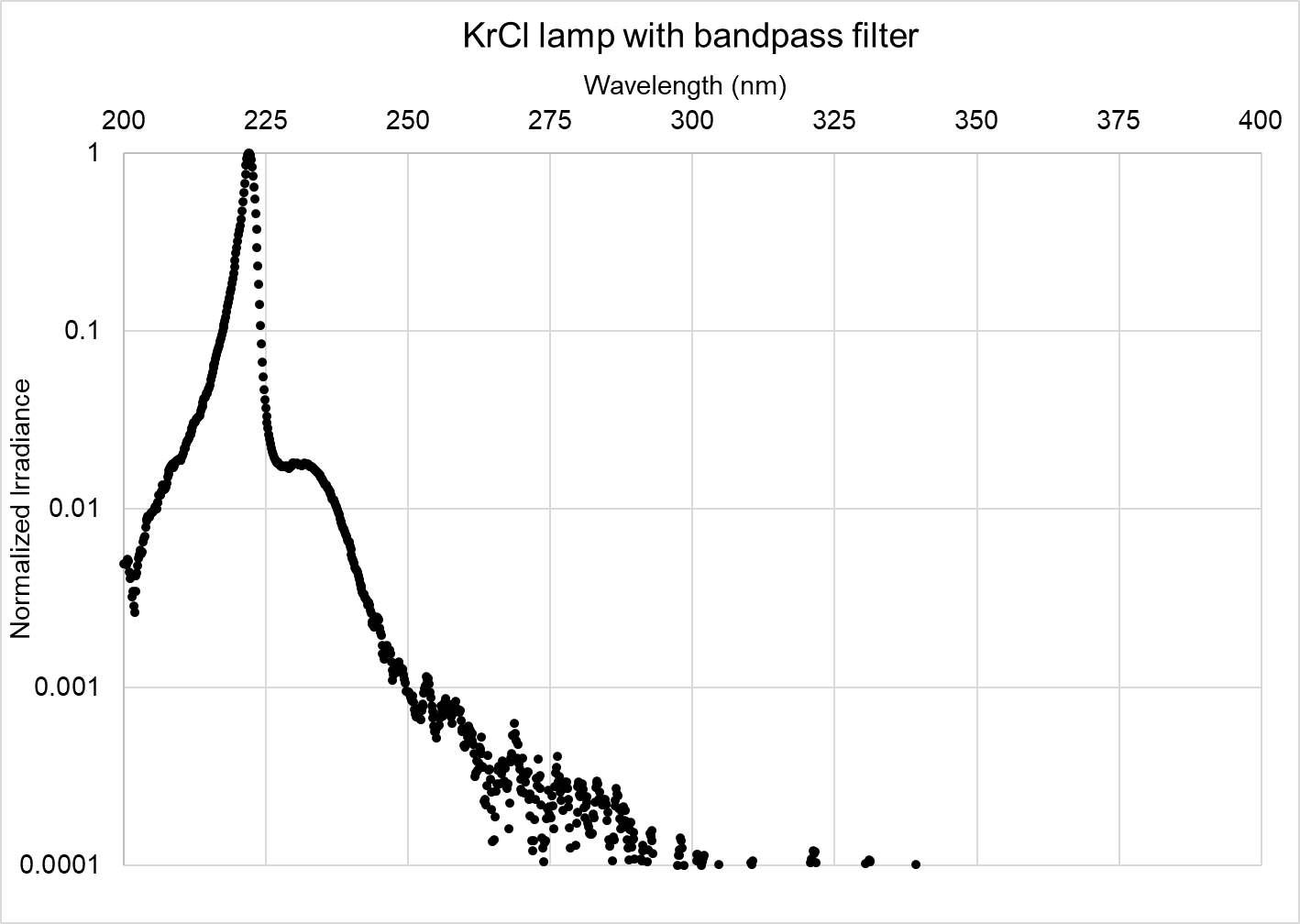


Figure S1. The normalized spectral irradiance of the KrCl lamp with the bandpass filter used in this study. Data was obtained using an Avantes AvaSpec-ULS4096CL-EVO Spectrometer.

Table S1. Data points for Figure 1.


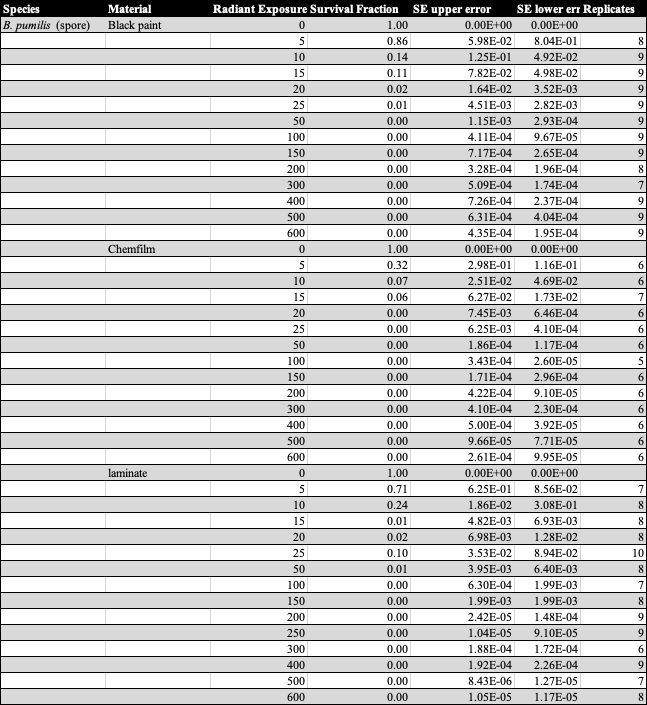


Table S1 cont.


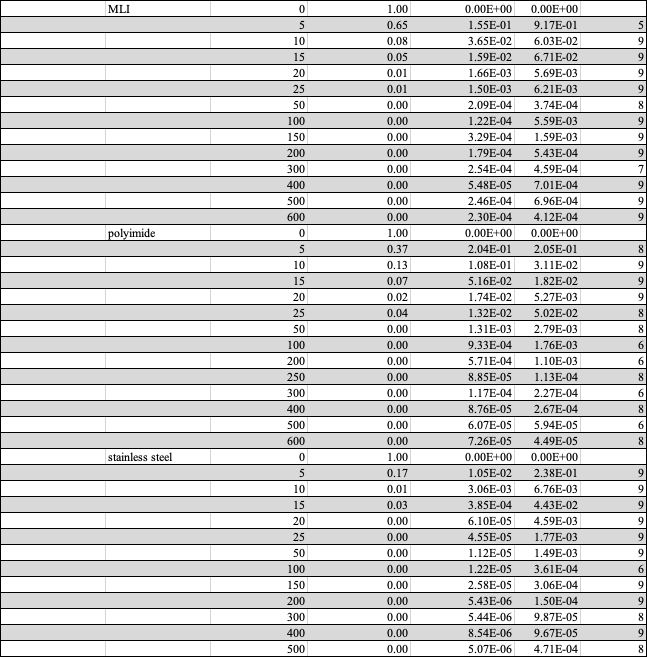


Table S1 cont.


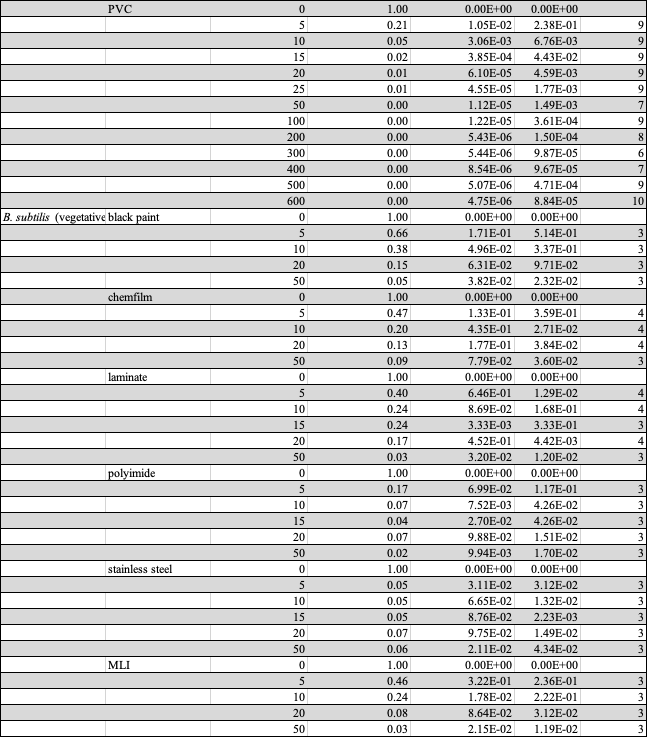


Table S1 cont.


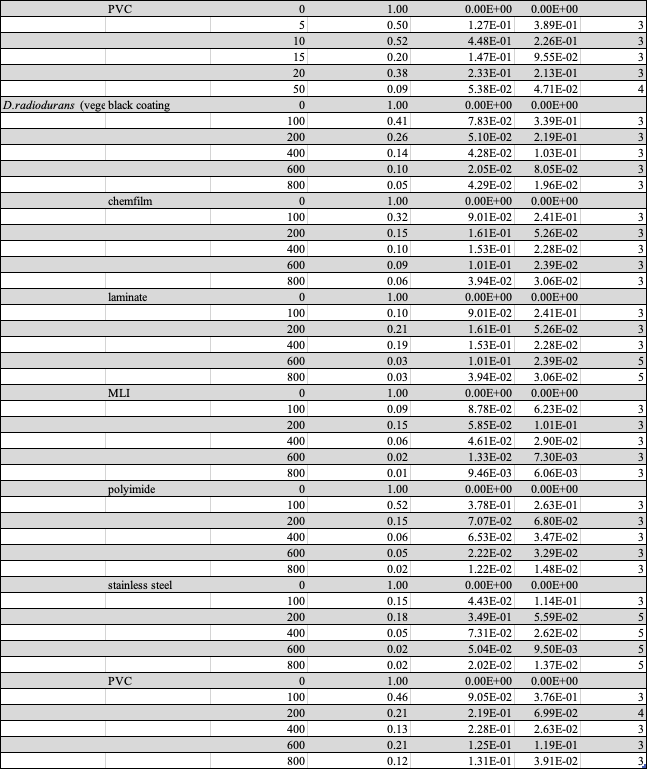

Supplement: Supplemental Material — Fig. S1 and Table S1. [file spectrum.02276-25-s0001.docx]
